# Real‐time monitoring of carbonation of hardened cement pastes using Raman microscopy

**DOI:** 10.1111/jmi.13084

**Published:** 2022-02-02

**Authors:** Kai Zhang, Marcus Yio, Hong Wong, Nick Buenfeld

**Affiliations:** ^1^ Department of Civil and Environmental Engineering Centre for Infrastructure Materials, Imperial College London London England

**Keywords:** calcium carbonate, carbonation, cement, concrete, non‐destructive testing, Raman

## Abstract

This study investigated the feasibility of Raman microscopy for monitoring early surface carbonation of hardened cement pastes in real time for up to 7 days. Samples were exposed to natural carbonation (440 ppm CO_2_) and accelerated carbonation (4% CO_2_), and the evolution of calcium carbonate (CaCO_3_) polymorphs, portlandite, ettringite, C‐S‐H gel and unreacted cement particles was followed. Results showed that calcite is the main polymorph formed under both natural and accelerated carbonation. Under accelerated carbonation, the formation of calcite on the sample surface completed within 1 day whereas under natural carbonation, the formation of calcite is expected to continue beyond 7 days. The contents of portlandite and ettringite decreased rapidly under accelerated carbonation but much more gradually under natural carbonation. However, calcium silicate minerals in unreacted cement particles remained unchanged throughout the carbonation processes. Overall, this study demonstrated that Raman microscopy is a valuable tool for non‐destructive real‐time imaging of surface carbonation in cement‐based materials.

## Abstract 2

Carbonation of cement‐based materials involves atmospheric CO_2_ reacting with calcium‐bearing phases to form calcium carbonate. Carbonation is an important process because it can affect the durability of concrete structures by enabling corrosion of steel reinforcement. It is also an important process for developing carbon capture, utilisation and storage strategies with cementitious materials. The ability to observe and measure carbonation in real‐time is therefore highly valuable. However, all of the commonly used methods are destructive and unsuitable for real‐time monitoring. In this study, we showed that Raman spectroscopy combined with confocal microscopy is a powerful non‐destructive technique for monitoring early surface carbonation and phase evolution during natural and accelerated carbonation of cementitious materials. Furthermore, the technique is applicable to rough surfaces in ambient environment, and is therefore suited for studying samples in their natural state. Raman microscopy is able to capture high‐resolution images of phase distribution at the microscale, providing interesting new insights into the mechanisms of carbonation.

## 1 INTRODUCTION

Carbonation in cement‐based materials involves the reaction between atmospheric CO_2_ and calcium‐bearing constituents, mainly calcium hydroxide (CH) and calcium silicate hydrate (C‐S‐H), to form calcium carbonate (CaCO_3_).^1^ Carbonation leads to a pH drop in concrete, thereby depassivating any embedded rebar in the carbonated zone and enabling corrosion. Natural carbonation of concrete is usually a very slow process, which can take decades to penetrate the concrete cover. Therefore, accelerated exposure involving high CO_2_ concentrations (1%–100%)^2^, ^3^, ^4^, ^5^, ^6^, ^7^, ^8^, ^9^ are often used in laboratory. Accelerated tests have been criticised as being unrepresentative of natural carbonation as the decalcification processes involved and the carbonates that formed tend to be different.^4^, ^10^, ^11^, ^12^ Nevertheless, accelerated carbonation is relevant to research on developing carbon capture, utilisation and storage (CCUS) strategies such as accelerated CO_2_ curing of concrete and carbonation of waste concrete fines. The ability to observe carbonation in real‐time would therefore be highly valuable to provide insights into the actual processes involved.

The most widely used method to investigate carbonation in concrete is the phenolphthalein spray test. Despite its simplicity and convenience, this technique is often criticised as underestimating the true carbonation extent in concrete due to its dependence on colour change that occurs when pH drops to < 9.5.^8^ Moreover, phenolphthalein is classified as a carcinogen and it could be phased out for future use to be replaced by thymolphthalein. Advanced techniques including thermogravimetric analysis, X‐ray diffraction (XRD) and infrared spectroscopy have been adopted to provide more accurate measurements of CaCO_3_ formed and therefore the extent of carbonation.^13^, ^14^, ^15^, ^16^, ^17^, ^18^ NMR could be used to detect amorphous CaCO_3_ that forms during early carbonation prior to crystallisation into vaterite and calcite. However, these techniques are destructive as they require substantial sample preparation including cutting, crushing and powder grinding, which may induce additional carbonation.^7^ The presence of aggregate particles in concrete further complicate data analysis.

Backscattered electron microscopy (BSE) has been widely applied to study microstructural changes induced by carbonation in concrete.^11^, ^16^, ^19^, ^20^, ^21^ However, as CaCO_3_ cannot be readily distinguished from other solid hydration phases, porosity change is often used as an indicator to evaluate the extent of carbonation.^22^ Micro‐tomography is another technique used to study changes in porosity as an indicator for carbonation. Although energy or wavelength‐dispersive X‐ray spectroscopy can be used to detect CaCO_3_ to some extent, the type of polymorphs formed cannot be determined.^23^, ^24^ Some techniques (e.g. SEM‐BSE) requires samples to be fully dried, epoxy‐impregnated, ground and polished, and therefore cannot be used for real‐time monitoring purposes.

Raman spectroscopy provides a non‐destructive means to characterise the phase composition of a sample based on molecular vibrations. When combined with confocal microscopy, the technique enables high resolution Raman imaging of phase distribution at a microstructural level. Furthermore, the technique can be applied to wet and uneven surfaces and therefore is suited for observing samples in their natural states. Raman microscopy has been used to study the hydration of C_3_S,^25^ weathering of anhydrous cements,^26^ and carbonation of lime paste.^27^ However, its application for real‐time imaging of cement‐based materials remains under‐explored. Torres‐Carrasco et al.^28^ and Mikhailova et al.^29^ used Raman microscopy to study the hydration of Portland cement and polymerisation of alkali‐activated cement and their results show that Raman microscopy is a promising tool for in situ observation of phase evolution in cement‐based materials.

The aim of this study is to determine the viability of Raman microscopy as a non‐destructive technique to monitor early surface carbonation and to study the phase evolution that occur during natural and accelerated carbonation. Hardened CEM I cement pastes exposed to continuous carbonation at 440 ppm and 4% CO_2_ levels were analysed at designated time points up to 7 days. The evolution of CaCO_3_ polymorphs, portlandite, ettringite, C‐S‐H gel and unreacted cement particles were analysed qualitatively and quantitatively using the collected Raman maps and spectra. The findings showed that Raman microscopy is a powerful tool for real‐time monitoring of carbonation in cement‐based materials, and provided some interesting new insights into the mechanisms involved. Further work to develop the technique is ongoing.

## 2 EXPERIMENTAL

### 2.1 Materials and sample preparation

Cement paste with a water/cement ratio (w/c) of 0.45 was prepared. Ordinary Portland cement CEM I 52.5N, with mineral composition of 62.4% C_3_S, 10.3% C_2_S, 10.6% C_3_A, 2.2% C_4_AF and 4.9% calcite was used. The specific gravity and loss on ignition were 3.06 and 2.1, respectively. The cement was mixed with tap water in a Hobart mixer for 3 min, and then compacted in two layers in a steel 50 mm cube mould. Immediately after casting, the sample was covered with plastic sheet and wet hessian for 24 h. Following that, it was demoulded and cured in a conditioning box at 20°C and 95% relative humidity (RH) for 3 days. After curing, the cubic sample was diamond sectioned vertically at the centre to produce two adjacent blocks (50 × 21 × 8 mm), one for natural carbonation and the other for accelerated carbonation. Prior to carbonation, the blocks were conditioned at 21°C, 65% RH in the presence of soda lime (CO_2_ < 10 ppm) for 1 day to stop hydration at the surface of the sample, and then viewed with SEM‐BSE (Hitachi TM4000, Tokyo, Japan) at 500× magnification to identify a matching region of interest (ROI) for analysis.

### 2.2 Carbonation

Carbonation of the block samples was carried out in two separate incubators (Panasonic MCO‐230ACI‐PE, Osaka, Japan), one maintained at 440 ppm CO_2_ for natural carbonation and the other at 4% CO_2_ for accelerated carbonation. The temperature and RH of the incubators were controlled at 21°C and 65%, respectively. The samples were exposed to carbonation in all directions. At each designated time point (0, 1 and 5 h, 1, 2, 3, 4 and 7 days), the samples were removed from the incubators and analysed with Raman microscopy. Each analysis took around 30 min and the samples were returned to their respective incubator immediately after analysis. Soda lime was placed in the microscope enclosure to minimise ambient carbonation during analysis.

### 2.3 Raman microscopy

Raman mapping was performed with a Renishaw inVia Qontor confocal microscope (Wotton‐under‐Edge, UK) at ambient conditions (21°C, 40% RH, 440 ppm CO_2_). Measurements were made using a laser line of 532 nm, grating of 2400 lines/mm and a long‐working distance objective of 50× magnification (NA = 0.5). The theoretical spot size of the laser was ∼1.3 μm. A thermoelectrically cooled charge‐coupled device (CCD) with 1024 × 256 pixels was used as the detector. Raman shift calibration was carried out using an internal silicon standard (520.2 ± 0.5 cm^–1^) prior to each scan. The laser power at the sample surface and exposure time were set to 1.25 mW and 0.5 s, respectively, to minimise laser‐induced damage.^30^ The spectral range was set from 143 to 1385 cm^–1^ to cover the main peaks of various CaCO_3_ polymorphs including calcite (280 and 1085 cm^–1^), aragonite (205 and 1085 cm^−1^) and vaterite (300, 1080 and 1090 cm^−1^).^31^ A total of 4675 points at a step size of 2 μm were analysed to give a Raman map of 18700 μm^2^. All data acquisition were performed using the Renishaw WiRE v.5.2 software. Spectral artefacts including cosmic rays and background were removed using the nearest neighbour method and least squares polynomial fit, respectively.

The Raman maps acquired were further quantified by image analysis to measure the volume fractions of calcite, portlandite and unreacted cement (C_2_S + C_3_S). Image segmentation was performed by manual thresholding. Since the ROIs analysed for natural and acceleration carbonation were not exactly the same, the quantified areas of the individual phases were normalised to that of the hydration product matrix (where carbonation mainly occurred) to enable a meaningful comparison.

## 3 RESULTS

### 3.1 Raman maps

**FIGURE 1:**
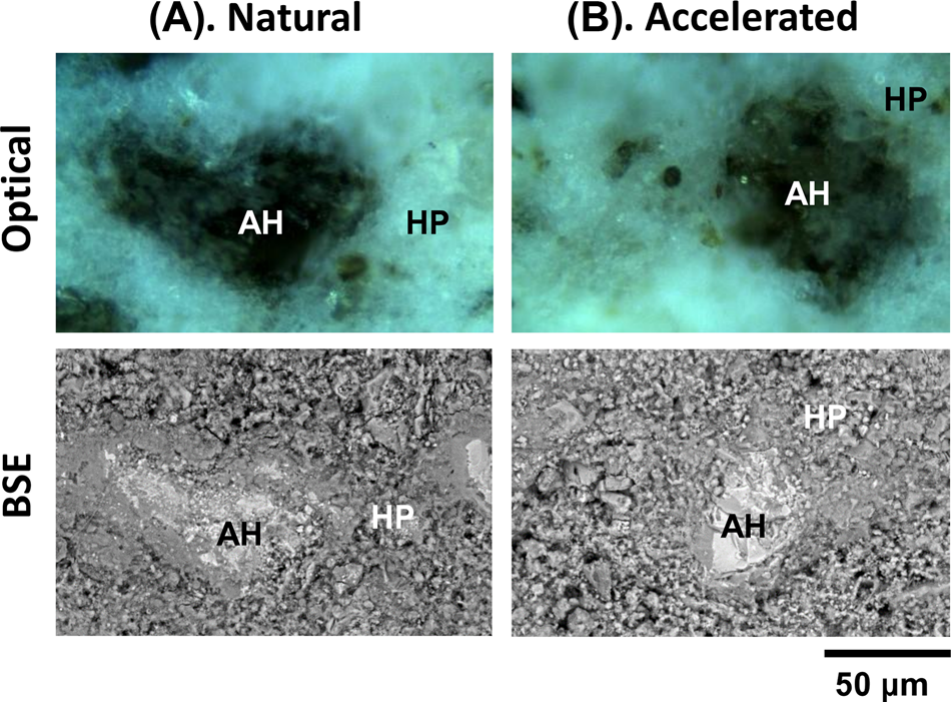
Area‐matching optical and BSE micrographs of the selected ROIs for Raman analysis to be exposed to (A) natural and (B) accelerated carbonation. AH: unreacted cement; HP: hydration product

Figure 1 shows the ROIs on each sample block selected for Raman mapping. Although the blocks were cut adjacent to each other, their microstructure appeared slightly different due to material loss caused by cutting. However, as confirmed by BSE, both ROIs contained a large unreacted cement particle (AH) surrounded by a porous matrix of hydration products (HP).

**FIGURE 2:**
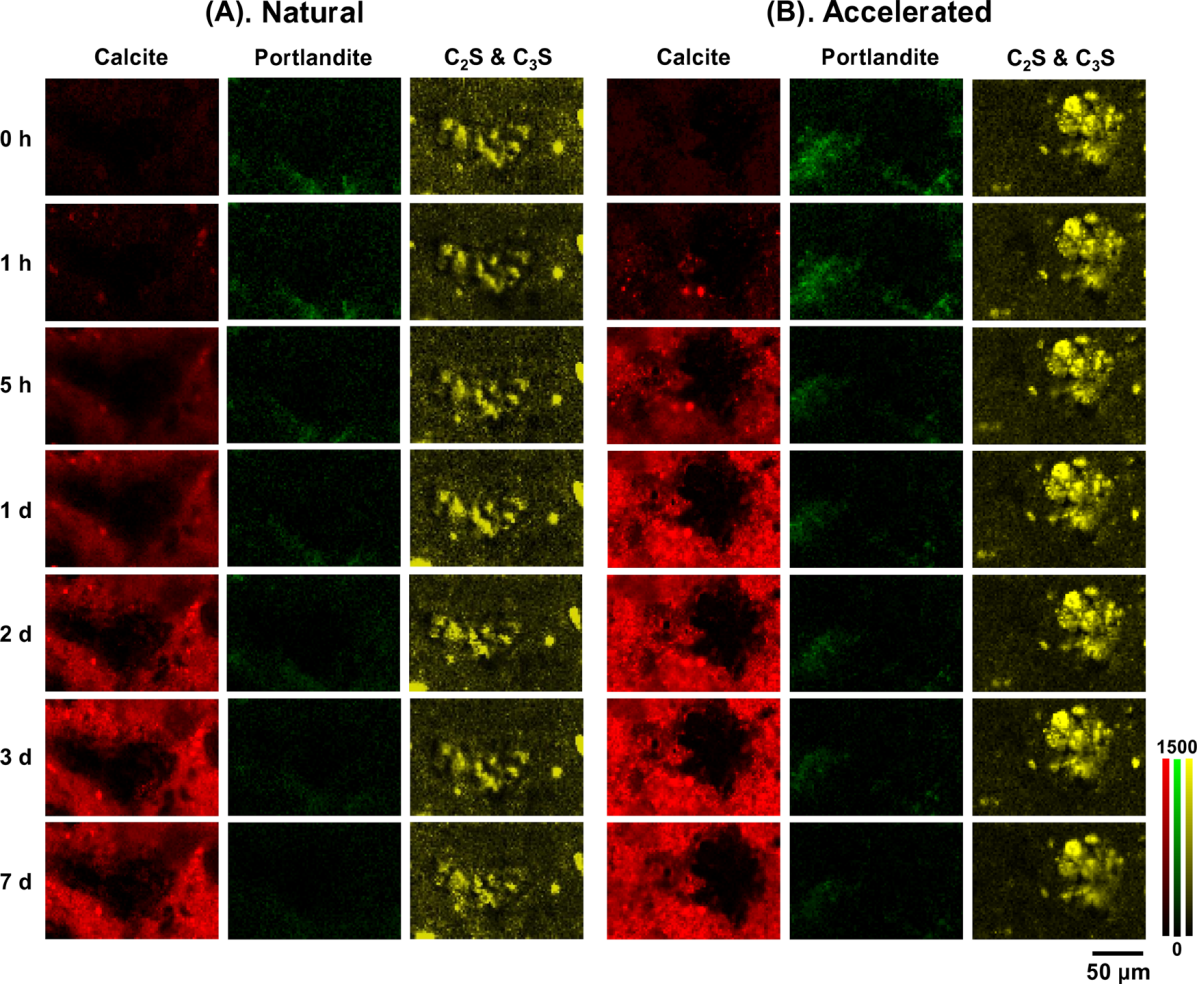
Raman maps showing the evolution of calcite, portlandite and C2S & C3S under (A) natural and (B) accelerated carbonation

Figure 2A and B presents Raman maps showing the evolution of calcite, portlandite and unreacted cement (C_2_S and C_3_S) under natural and accelerated carbonation, respectively. The maps were produced based on the intensity of the main Raman peaks, that is 1085 cm^–1^ for calcite, 356 cm^–1^ for portlandite, 877 cm^–1^ for C_2_S and 835 cm^–1^ for C_3_S.^28^ Dark pixels indicate low content whereas bright pixels indicate high content of the respective phase. However, it should be pointed out that pixel brightness is also dependent on the crystallinity of the phases^28^ such that more crystalline phases (e.g. calcite) tend to produce higher signals and hence brighter pixels.

At 0 h, no calcite (or other polymorphs of CaCO_3_) was detected in either sample, confirming that carbonation had not occurred during curing or conditioning. The calcium silicates present within the unreacted cement particles show relatively well‐defined morphology, with the remaining areas likely to be surface irregularities. Small amounts of portlandite were observed in both samples at 0 h although the signals were relatively weak. Between 1 and 5 h of carbonation, calcite began to appear in both samples in the hydration product matrix. This confirms that carbonation occurs very rapidly on concrete surface even if it takes place naturally under ambient conditions. However, the amount of calcite formed during accelerated carbonation was clearly more than that of natural carbonation.

After 1 day, the surface of the sample exposed to accelerated carbonation appeared fully carbonated, evident by the consistent pixel brightness of calcite. This suggests that carbonation had completed at the sample surface after 1 day and had possibly advanced into the subsurface. In contrast, the surface of the sample exposed to natural carbonation showed gradual increase in calcite concentration and the data suggest that the formation of calcite will continue beyond 7 days. In either case, the intensity of the portlandite signal decreased with time, indicating that portlandite had reacted with CO_2_ to form CaCO_3_. However, calcite did not only form where the portlandite was located but also elsewhere in the cement paste matrix due to dissolution‐precipitation process. Some C‐S‐H gel and perhaps also ettringite might have also carbonated to form calcite.^32^ However, this was not evident from the Raman maps due to the very weak signals of these phases (hence not presented). The unreacted cement particles remained relatively unaffected throughout the exposure to CO_2_. However, it is expected that with a longer period of carbonation, unreacted cement can also become decalcified.^33^, ^34^

### 3.2 Raman spectra

**FIGURE 3:**
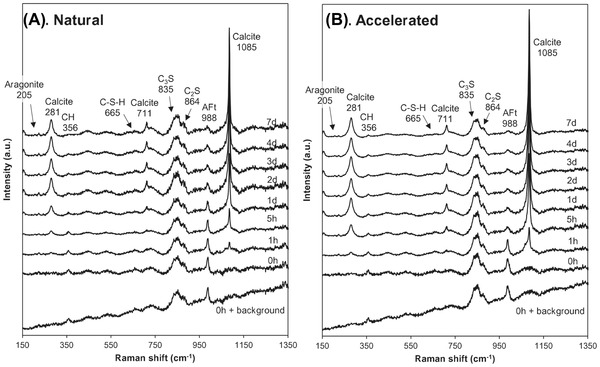
Raman spectra showing the evolution of the peaks of different phases under (A) natural and (B) accelerated carbonation

The Raman spectra from each map were summed up and compared to observe all detectable phases. Results are presented in Figure 3. In corroboration with Figure 2, the intensity of the Raman peak for calcite (1085 cm^–1^) increased over time and advanced more rapidly under accelerated carbonation. Correspondingly, the intensity of the Raman peak for portlandite (356 cm^–1^) decreased with carbonation. Other polymorphs of CaCO_3_ including aragonite (205 cm^–1^) and possibly vaterite (300 cm^–1^) were also detected, especially under accelerated carbonation, but their signals were very weak. These polymorphs are known to be metastable and act as precursors to the precipitation of more stable calcite.^21^, ^35^. It should be pointed out that amorphous calcium carbonate can also form prior to the formation of crystalline CaCO_3_.^36^ Indeed, at 1 h carbonation, a hump attributed to the symmetric stretching of C‐O bonds of different lengths^37^ was observed between 1080 and 1090 cm^–1^ in addition to the crystalline peak at 1085 cm^–1^ and a weak lattice peak at 281 cm^–1^. It was therefore likely that both amorphous and crystalline CaCO_3_ coexisted in the sample at early carbonation. In addition to amorphous calcium carbonate, monohydrocalcite is another hydrated form of CaCO_3_ ^38^ but this was not observed (1069 cm^–1^)^39^ in this study.

The characteristic Raman peak of C‐S‐H (665 cm^–1^) was also detected but the signals were similarly very weak due to its poor crystallinity.^28^ However, careful observation suggests that C‐S‐H remained relatively unchanged under natural carbonation but slightly decreased with accelerated carbonation after 1 day. This appears to be consistent with the study by Castellote et al.^4^ who reported significantly enhanced decalcification of C‐S‐H under accelerated carbonation. Another detectable phase worth discussing is ettringite (988 cm^–1^). The presence of ettringite is unsurprising given the relatively young age of the sample. It is clear from the spectra that ettringite decreased with increasing carbonation, especially after 1 day of accelerated carbonation. It has been reported by Auroy et al.^32^ that pure ettringite can decalcify to form aragonite, gibbsite and either gypsum or bassanite under accelerated and natural carbonation, respectively, but the Raman peaks for these phases were not detected in this study.

### 3.3 Quantification by image analysis

**FIGURE 4:**
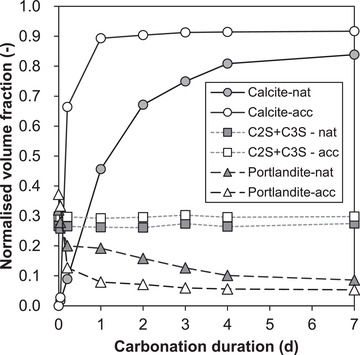
Evolution in the volume fractions of calcite, C2S + C3S and portlandite quantified by image analysis of the Raman maps

Image analysis results (Figure 4) show that under accelerated carbonation, nearly all calcite was formed in the first day, whereas under natural carbonation, the formation of calcite was much slower and likely to continue beyond 7 days. This corresponds with the trends of portlandite which appears to decrease rapidly under accelerated carbonation and much more gradually under natural carbonation. The volume fraction of C_3_S and C_2_S, in contrast, remained stable throughout carbonation in both exposure regimes. Overall, the results appear to corroborate the qualitative observations made in Sections 3.1 and 3.2.

## 4 DISCUSSION

This study demonstrates the feasibility of Raman microscopy for real‐time mapping of early carbonation in cementitious materials. The technique is non‐destructive and requires very minimal sample preparation, and therefore is advantageous for observing hydrated samples. The high spatial resolution also enables the distribution of various phases to be observed at a microstructural level. However, Raman mapping of large surface area can be time‐consuming and prolonged scanning may lead to microstructural changes as a result of drying, although this was not observed in the current study. This can be limited to some extent by keeping the laser power and exposure time low. Alternatively, an in situ environmental chamber may be used to maintain the atmosphere in the microscope and to avoid the need to remove the sample.

Further work is required to confirm the findings presented in this paper. For example, the analysis of multiple locations will improve the representativeness of the results. Other techniques including XRD, infrared spectroscopy and BSE‐EDS may also be used to provide additional supporting data, especially concerning phases that are less sensitive to Raman imaging such as C‐S‐H, vaterite and aragonite. Given that the depth of carbonation has practical significance, work is ongoing to adapt the technique for depth profiling to monitor sub‐surface changes and the progress of the carbonation front in cement‐based materials. This is inherently challenging to achieve in real‐time and without destroying the sample.

## 5 CONCLUSION

Raman microscopy was used to study the evolution of hardened cement pastes subjected to surface carbonation. The evolution of calcium carbonate polymorphs, portlandite, unreacted cement and other phases including ettringite under natural carbonation (440 ppm CO_2_) and accelerated carbonation (4% CO_2_) was investigated. Results showed that surface carbonation completed within 1 day of accelerated carbonation, but the formation of calcite is expected to continue beyond 7 days for natural carbonation. Portlandite and ettringite decreased rapidly under accelerated carbonation but much more gradually under natural carbonation. Unreacted cement particles remained relatively unaffected throughout the 7‐day exposure to carbonation. Overall, this study demonstrated that Raman microscopy is a powerful tool for non‐destructive real‐time monitoring of surface carbonation. Further work to apply the technique to monitor sub‐surface evolution and depth profiling of carbonation front is ongoing.
